# A dataset of pre-pandemic African protected area visitation

**DOI:** 10.1038/s41597-025-04998-7

**Published:** 2025-05-08

**Authors:** Falko Buschke, Claudia Capitani, Philipp Schägner, Christophe Nsengiyumva, Hellen Okelo, Oumar Cissé, Susan Snyman

**Affiliations:** 1 European Commission, Joint Research Centre, Ispra, Italy; 2 German Environment Agency, Dessau-Roßlau, Germany; 3 African Leadership University, School of Wildlife Conservation, Kigali, Rwanda; 4 School of Tourism and Hospitality, College of Business and Economics, University of Johannesburg, Johannesburg, South Africa; 5 Present Address: German Federal Statistical Office, Bonn, Germany

**Keywords:** Conservation biology, Ecosystem services

## Abstract

When people visit protected areas, their presence can amplify public support for conservation and their spending closes conservation finance gaps and feeds local economies. Across Africa, protected areas are often presented as engines for poverty alleviation and rural development. Yet visitation data remains scarce for most of the continent. Here we present a dataset of African protected area visitation obtained from government sources as well as peer-reviewed and grey literature. The spatially explicit dataset includes 4,216 records from 341 protected areas in 34 countries. The earliest visitor counts date back to 1965, but the majority (78%) stem from between the years 2000 and 2020. While 22% of protected areas only have visitation data for a single year, the median protected area has six years of visitation data, facilitating temporal analyses. Moreover, the dataset is compatible with the World Database of Protected Areas, making it possible to compare visitation across governance types and management categories. Ultimately, the dataset provides baselines for post-pandemic nature tourism recovery and enables analyses of the factors determining protected area visitation.

## Background & Summary

The number of visitors to African protected areas matters^1^. When people visit protected areas, they do not only pay entry fees that support conservation^2^. They also support employment opportunities^3–5^, and produce economic multipliers that permeate the broader region^6^. Visitors are a major source of foreign income^7,8^, and protected area tourism has been shown to be associated with more wealth, less poverty, and improved child health in neighbouring communities^9^. As a consequence, protected area visitation is inherently political when it attracts government budget allocations because visitors are seen as political supporters and regional spenders^10^.

Protected area visitation is central to continental science-policy discussions. For example, the African regional assessment by the Intergovernmental Science-Policy Platform on Biodiversity and Ecosystem Services (IPBES) outlines how governments stand to benefit from tourism income and employment when establishing protected areas^11^. Similar reasoning underlies the African Union’s Green Recovery Action Plan 2021–2027^12^, which aims to develop protected areas to revitalise ecotourism as a means to stimulate post-pandemic socio-economic recovery.

At the same time, protected area visitation is also presented as (i) a proxy for, (ii) a driver of, and (iii) a justification for effective protected area management. For example, when econometric studies presume that better managed areas attract more tourism^13^, visitation data is used as a proxy for effectiveness. For example, in Ghana poaching incidents declined with increased visitation^14^. By contrast, falling visitation rates and the associated drops in revenue – such as reductions caused by the Covid-19 pandemic – can undermine conservation efforts and erode confidence in current conservation models^15^. Here, visitation information is interpreted as a potential driver of future management effectiveness. Lastly, visitation data can justify conservation actions when conservation interventions are framed relative to impact on tourism revenue. For example, anti-poaching investments in African protected areas have been justified economically by the foregone tourism revenue caused by the illegal killing of elephants^16^.

Despite the economic, political and environmental value of protected area visitation, consolidated visitor information remains scarce for most African protected areas. More than 15 years ago, Balmford and colleagues compiled a global dataset of visitation to 280 protected areas in 20 countries^17^. This dataset included information from 43 protected areas across five African countries but was not released publicly. Six years later, this foundational dataset formed the framework for an expanded analysis^18^, which included publicly released visitation information from 71 protected areas across 16 African countries (not including visitation counts from the 23 Madagascan protected areas, which were not released publicly). The following year in 2016, summarised average annual visitation counts were published for a total 164 protected areas from 25 African countries^16^. While there have been more recent efforts to collate visitor data elsewhere in the world^19,20^, these studies did not upgrade visitor data for Africa. These pioneering efforts are invaluable but demonstrate the significant information gaps that remain.

**Fig. 1:**
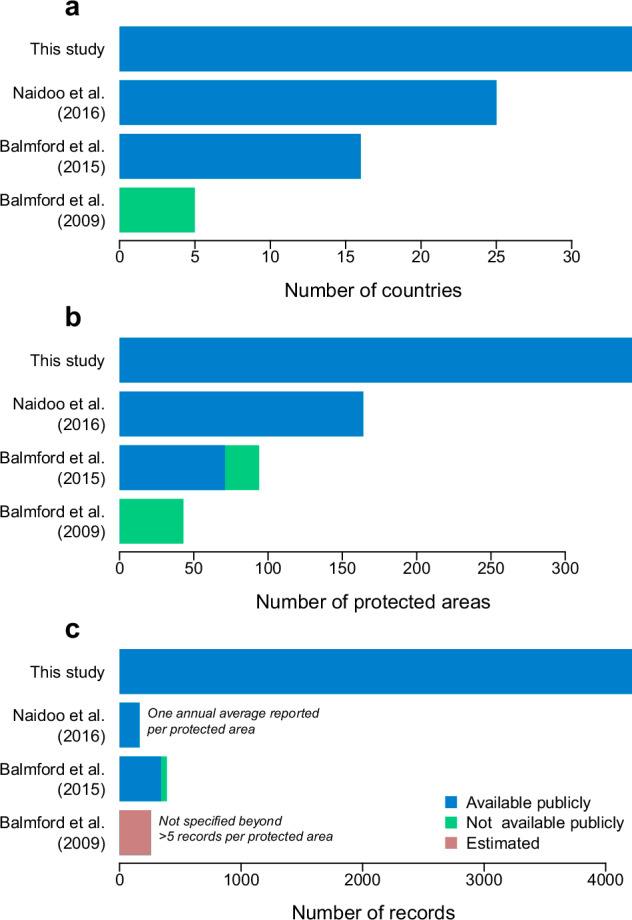
The dataset of African protected area visitation considerably expands existing information from prior studies. The dataset presented with this study includes data from more (**a**) African countries and (**b**) protected areas as well as (**c**) more annual records of visitation counts compared to prior synthesis studies by: Balmford *et al*. (2009)^17^, Balmford *et al*. (2015)^18^, and Naidoo *et al*. (2016)^16^. (The number of annual records was not specifically reported by Balmford *et al*. (2009) but because they only included protected areas with six or more annual visitor records in their study^17^ we obtained a lower estimate of the total number of records by multiplying the number of protected areas, (**b**), by six.).

Here we present a new consolidated dataset of protected area visitation in Africa. This dataset is a major advance on existing studies (Fig. 1) because it expands the temporal and geographical coverage of visitation data using a standardised – thus, comparable – format. A major feature of the dataset is its compatibility with the World Database on Protected Areas (WDPA)^21,22^. Cross-compatibility means that visitation data can be combined with the geographical attributes and related metadata of the WDPA (such as the age, area, governance type, or management category). Where available, visitation data are further enriched with supporting information of the type, method, and certainty of the visitor count; all aspects that influence how such data ought to be interpreted^1^. Ultimately, the dataset provides baselines for post-pandemic ecotourism recovery and enables analyses of the factors determining protected area visitation.

## Methods

### Backbone datasets

The dataset of protected area visitation started from publicly available data from two previously published studies^16,18^ (Fig. 1). We filtered data from African protected areas, reformatted the visitor counts, and checked for duplicates across the datasets. When the same protected area was reported across both studies, we only included the data from Balmford *et al*.^18^ because the data were disaggregated by year (unlike data from Naidoo *et al*.^16^, which averaged visitation counts over multiple years). Once the data were cleaned, we cross-checked each protected area with the WDPA^21^ to validate the names of each protected area and identify their World Database on Protected Areas Identifier Code (WDPAID), the unique identifier assigned to each protected area by United Nation Environment Programme’s World Conservation Monitoring Centre (UNEP-WCMC). This WDPAID was essential to identify the geographical location and related attributes for each protected area.

### Literature search

We supplemented the backbone dataset with visitor records from an online search of peer-reviewed and grey literature in 2021. Because we anticipated that most visitor information would be in the grey-literature (e.g., unreviewed reports by government ministries, protected area agencies, or NGOs), we followed a more flexible approach than recommended for typical systematic reviews of the peer-reviewed literature^23–25^. In addition to the Web of Science Core Collection (https://www.webofscience.com/wos/woscc/basic-search) of peer-reviewed research, we also searched Google (https://www.google.com/), Google Scholar (https://scholar.google.com/), and ResearchGate (https://www.researchgate.net/). We recognise the limitations of using the latter three data sources for replicable information searches and retrieval (i.e., these platforms use customised search algorithms that are tailored to the specific profile of the user and are, therefore, affected by prior search histories)^26–28^. However, our goal was to cast a wide net to target websites that may contain relevant information after subsequent manual inspections. Since our search was constrained to English sources, it likely underestimates public records from countries where English is not the official language.

We used variations of the following a generic search string by varying the country name or region:


["total annual visit*" OR "annual visit*" OR "visit* per year" OR "yearly visit*" OR "visit* statistic" OR "visit* data" OR "visit* number" OR "number of visit*" OR "visit* count" OR "visit* estimation" OR "visit* monitoring"] AND ["protected area" OR "national park" OR "conserv* area"] AND [{Country name} OR {Region name}]


Visitation data from the search items were added to the dataset^29–79^. In instances where the document search identified visitation counts for the same protected areas already represented in the backbone datasets, we manually checked whether visitation counts were consistent across the sources. If there were any disparities, we only included the visitation counts from the peer-reviewed backbone datasets^16,18^. Each protected area added from the online search was cross-validated with the WDPA^21^ to confirm the name and WDPAID of the protected area.

### Country outreach

**Fig. 2:**
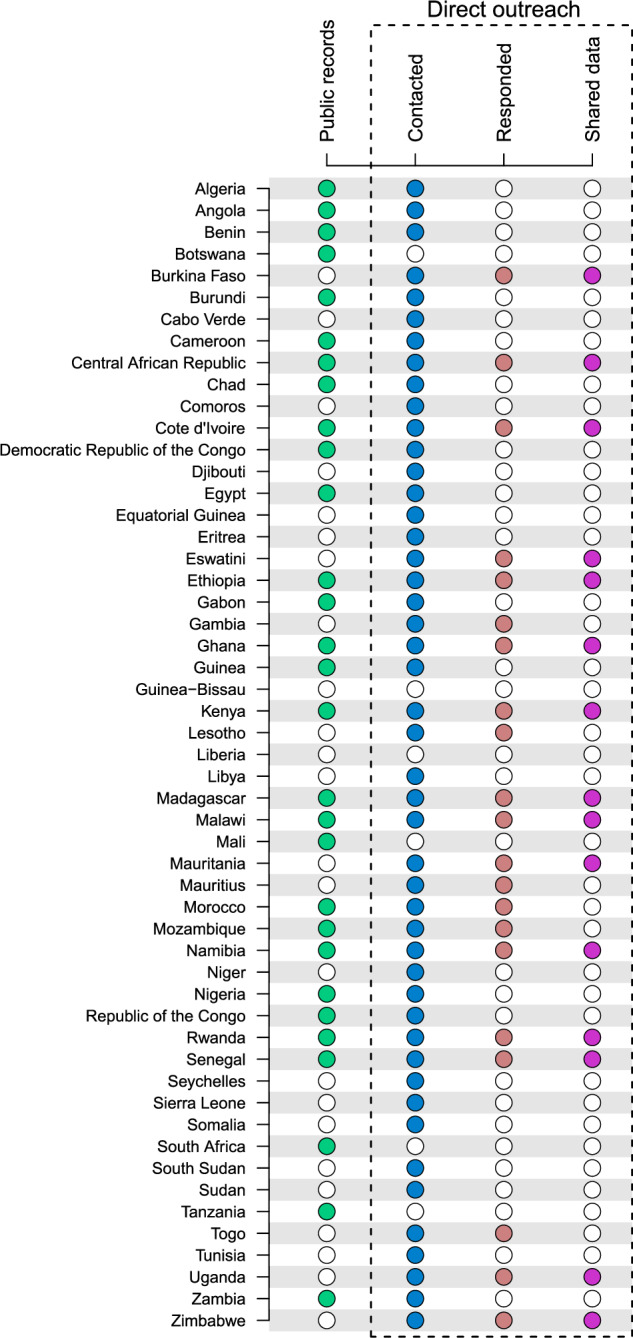
A summary of how protected area visitation data were obtained for each African country. Data either were from public records (i.e., peer-reviewed or grey literature for 29 countries) or sourced directly from outreach to country representatives. Forty-seven country representatives were contacted using email, of which 21 responded to messages and 15 shared visitation data.

We obtained protected area visitation data from 29 out of 53 African countries from the backbone published searches and the online search (Fig. 2). Using email, we contacted national representatives from 47 African countries between June and September 2021. Of the six countries that were not contacted, three countries (Botswana, South Africa, and Tanzania) were deprioritised because we had already sourced visitation data from public sources, and a further three countries (Guinea-Bissau, Liberia, Mali) could not be contacted because we were unable to find appropriate contact information. For the remaining countries, 77% of contacts were from government ministries, 17% from park authorities, and 6% were from academia (we do not share more detailed contact information here to maintain the privacy of respondents, particularly those that did not respond to our messages). Twenty-one country representatives (40% of all African countries) responded to our email requests, of which 15 (29% of all African countries) shared visitation data that were incorporated into the dataset (Fig. 2). If country representatives shared visitation data for protected areas that were already in the dataset, we checked for consistency across the different sources and, if we found any disparities, we favoured the information provided by the national representatives. Each protected area added from country outreach was cross-validated with the WDPA^21^ to confirm its name and WDPAID.

## Data Records

### Overview of data structure

The dataset of African protected area visitation contains 4,216 visitation records from 341 protected areas in 34 African countries, which represents a substantial increase relative to existing information from prior studies (Fig. 1). These data are publicly available as a single file in.csv format from Zenodo^80^. The dataset has 29 columns containing the following information:

- **ID** (included for 100% of records): A unique integer identifier code for each record. The order of the identifier codes is arbitrary and the sequence is not reflective of visitation characteristics.
- **Country** (included for 100% of records): The name of the country from which the record originates.
- **PA_name** (included for 100% of records): The name of the protected area, consistent with the name as recorded by the WDPA^21^.
- **WDPAID** (included for 100% of records): A unique identifier assigned to each protected area by UNEP-WCMC as part of the WDPA^21^. For 7.9% of protected areas not included in the WDPA (e.g., botanical gardens and wildlife sanctuaries), arbitrary identifiers were assigned using the prefix XID followed by a unique integer (e.g., XID1, XID2, …, XID99). The prefix ensures that the dummy codes cannot be mistaken for WDPAIDs. Overall, these dummy codes affected 5.5% of all records.
- **IUCN_Cat** (included for 94.5% of records): The IUCN Management Category^81^ as reported by the WDPA^21^. Only 10 levels permitted Ia (Strict nature reserve); Ib (Wilderness area); II (National Park); III (Natural monument or feature); IV (Habitat or species management area); V (Protected landscape or seascape); VI (Protected area with sustainable use of natural resources); Not Applicable; Not Assigned; Not Reported.
- **Longitude** (included for 100% of records): The longitude of the internal geographical centroid of the protected area.
- **Latitude** (included for 100% of records): The latitude of the internal geographical centroid of the protected area.
- **REP_AREA** (included for 94.5% of records):The area in square-kilometres as reported by the data suppliers to WDPA^21^.
- **GIS_AREA** (included for 94.5% of records):The area in square-kilometres calculated by UNEP-WCMC using GIS from protected area polygons in the WDPA^21^. The GIS areas of protected areas in the WDPA only represented by points were reported as 0 km^2^.
- **STATUS_YR** (included for 94.5% of records):The age of the protected area, as inferred from the year in which the most recent status (e.g. proposed, designated, established etc.) was enacted according to the WDPA^21^. The WDPA reported the status year as 0 for 2.8% of records.
- **GOV_TYPE** (included for 94.5% of records):The type of governance^82^ in the protected area according to the WDPA^21^. Only 12 categories permitted: Federal or national ministry or agency; Sub-national ministry or agency; Government-delegated management; Transboundary governance; Collaborative governance; Joint governance; Individual landowners; Non-profit organisations; For-profit organisations; Indigenous peoples, Local communities; Not Reported.
- **ISO** (included for 100% of records): The 3-letter country code as defined by the International Organization for Standardisation.
- **Visitors** (included for 100% of records): The count of visitors to the protected area.
- **Year** (included for 100% of records): The year of the visitation count
- **VisitationType** (included for 97.5% of records):A description on the type of visitor count^1^ in one of eight categories: Entrants, International visitors; Reported Averages; Sum of international and domestic visitors; Unknown/other, Visitors; Visits.
- **International_tot** (included for 14.2% of records): The total count of international visitors to the protected area.
- **International_perc** (included for 14.2% of records): The number of international visitors as a percentage of the total number of visitors.
- **ForeignResidents_tot** (included for 1.3% of records): The total count of foreign resident visitors to the protected area.
- **ForeignResidents_perc** (included for 1.3% of records): The number of foreign resident visitors as a percentage of the total number of visitors.
- **Domestic_tot** (included for 13.5% of records): The percentage of domestic visitors to the protected area.
- **Domestic_perc** (included for 13.5% of records): The number of domestic visitors as a percentage of the total number of visitors.
- **Spending** (included for 0.8% of records): The estimated total spending by all visitors.
- **Currency** (included for 0.8% of records): The currency of the spending estimates.
- **Int_US$** (included for 0.8% of records): Spending presented in International US$ according exchange rates in September 2024.
- **Method** (included for 50.9% of records): The method used to quantify visitation. Four levels: Accounting data; Permit sales; Unknown/other; Visitor questionnaires.
- **Confidence** (included for 97.6% of records): The confidence that the visitation count represents the reported figures accurately (i.e., this is not a certainty estimate of the counts themselves). Currently five levels: unknown; speculative; moderate; high; very high
- **Comments** (included for 9.0% of records): Any comments that may affect the interpretation of the visitation counts.
- **Reference_original** (included for 100% of records): The reference to the original data.
- **Citation_Comment_Sept2024** (included for 100% of records): A comment to validate whether the original data source is still available.

### Summary of data attributes

**Fig. 3:**
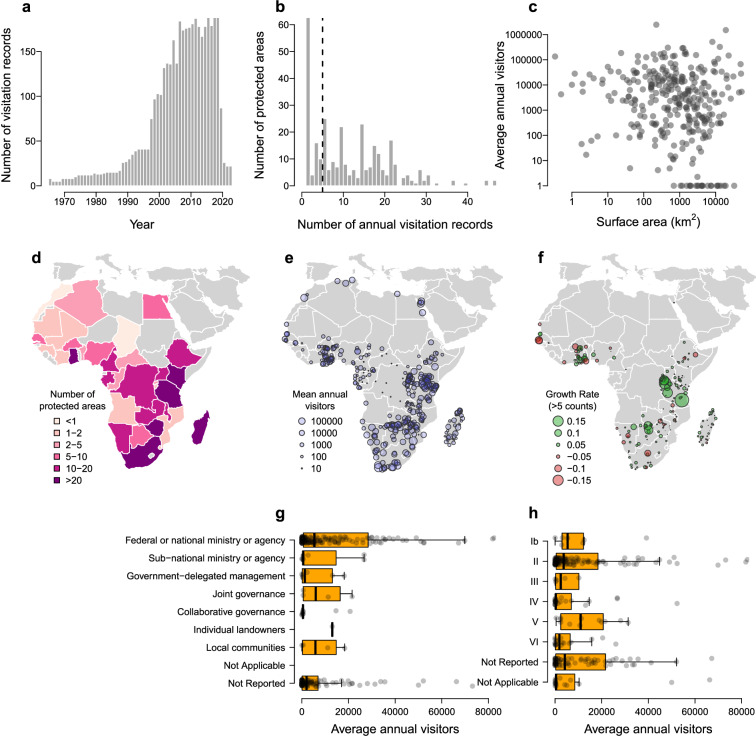
A summary of information in the dataset of African dataset of protected area visitation. (**a**) Although the earliest records in the dataset are from 1965, 78% of records are from the period 2000–2020. (**b**) The median protected area contains six years of visitation counts, though 22% of protected areas only have a single visitation record. The dashed vertical line shows the median of six visitor counts per protected area. (**c**) Mean annual visitation is not related to the surface area of protected areas (note, both axes are log-transformed). (**d**) Data are available from 34 African countries, with highest protected area coverage in Madagascar, Ghana, Zimbabwe, South Africa, and Tanzania. (**e**) The mean annual visitation to individual protected areas, showing higher visitation in Eastern and Southern Africa. (**f**) Trends in visitation growth rates for protected areas with more than six or more annual visitation counts. The average annual visitation to protected areas with different (**g**) governance and (**h**) IUCN management categories.

The earliest visitation records in the dataset are from 1965, the most recent from 2023, and 78% of records are from the period 2000–2020 (Fig. 3a). The median protected area in the dataset had six visitation counts (vertical dashed line in Fig. 3b). Roughly one out of five (22%) protected areas only had visitor counts from a single year (Fig. 3b), but four Kenyan protected areas (Aberdare, Mount Kenya, Tsavo East, and Tsavo West) had 55 years of continuous visitation data between 1965 and 2019. The surface areas of protected areas in the dataset are right-skewed, with relatively fewer large areas, though there is no clear relationship between visitation and the geographical extent of protected areas (Fig. 3c). The smallest protected areas in the dataset with reliable area information are less than 0.5 km^2^ (Kisumu Impala Sanctuary, Kenya; Popo Game Park, Namibia), while the largest covers an area of 52,800 km^2^ (Central Kalahari Game Reserve, Botswana).

Visitation data are available for 34 African countries (Fig. 3d), from a median of seven protected areas per country. Madagascar (33 protected areas), Ghana (30 protected areas), Zimbabwe (25 protected areas), South Africa (24 protected areas), and Tanzania (24 protected areas) have data for the most protected areas. Data are generally lacking throughout North Africa, the Sahel and the Horn of Africa, and parts of West Africa.

Average annual visitation varied across the continent, with most protected areas receiving few visitors each year (median annual visitation = 2,726 visitors) (Fig. 3e). A third of protected areas (33.0%) received more than 10,000 visitors per year, and 7.8% received more than 100,000 visitors per year. Only two protected areas received more than one million visitors per year on average (Table Mountain National Park = 2.4 million; Kruger National Park = 1.5 million, both in South Africa). Protected areas in southern and eastern Africa received more visitors, with the following countries ranked by average visitation per protected area: South Africa, Kenya, Tanzania, Zimbabwe, Egypt, Namibia.

Trends in visitation rates (Fig. 3f) can be estimated using the same approach as Balmford and colleagues^17^, where a linear regression model is fit to protected areas with six or more visitation records, and then dividing the slope parameter the maximum number of visitors to estimate a rate of change standardised between −1 and +1. Growth rates estimated in this way were normally distributed (median = 0.016), with the largest declines in Réserve Naturelle Communautaire de Palmarin (−0.11), Senegal, and the greatest increases in in Nyerere National Park (+0.2), Tanzania.

Because the dataset is compatible with the WDPA, visitation data can be disaggregated by IUCN governance type (Fig. 3g) and IUCN management category (Fig. 3h). Given that data were partly collected from national authorities, federal or national government ministries or agencies were reported to govern 52.6% of protected areas in the dataset. Similarly, 43.1% of the protected areas were managed Category II protected areas (i.e., National Parks).

## Technical Validation

The dataset of African protected area visitation is based on secondary data sources, so we were unable to directly validate the original visitor records for each protected area. We, therefore, assumed that the visitation records – whether published or provided to us by national representatives – were accurate. However, we ensured the reliability of the consolidated dataset using four strategies.

First, different individuals collected and consolidated the visitor data to remove observer bias. Data were initially entered into separate collection forms, and then these data were copied to a new file before they were combined with data from other forms. This two-stage process meant that any data entry errors were identified prior to being added to the consolidated dataset.

Second, we recorded the original source of visitation data in the column Reference_original. Users are then able to trace the origin of each data record. Moreover, each reference was manually double-checked, and the accuracy of the original reference was recorded in the column Citation_Comment_Sept2024.

Third, each record was manually cross-checked with the WDPA^21^, to confirm that the name and identifier code (WDPAID) of the protected area was correct. While this step did not validate the visitation count, it ensured that the associated metadata were consistent with the leading global standard^22^.

Fourth, every data record was checked (both manually and programmatically) to remove duplicates. The remaining data were plotted (including Fig. 3) and inspected for outliers or errors. Any artefacts identified in the data are recorded transparently in the Comment column of the dataset.

## Usage Notes

The dataset is a single standalone.csv file, which can be accessed using most text-readers, spreadsheets, or statistical software. However, users may find it helpful to re-use our code developed for R version 4.4.0^83^ available from Zenodo^80^ to transform, summarise, and display the data.

For geographical analyses, we strongly recommend that users obtain the latest version of the WDPA^21^, and perform a spatial join between the WDPA and the African protected area visitation dataset using the shared WDPAID attribute. The WDPA is updated periodically, so users should ensure that they are using the latest version of the protected area shapefiles. This is particularly relevant if the aim is to evaluate protected area visitation relative to geographical confounding variables (e.g., natural characteristics, protected area isolation, human population density, or national economic production^18^).

For comparative analysis or economic modelling, users ought to be aware of the different ways of quantifying protected area visitation^1^, which is described in the column VisitationType in the dataset. For example, although 83% of records are for *visitors*, approximately 4% of records account for *visits* (i.e., if the same person enters and exits the same protected area multiple times a day, it would count as a single *visitor*, but multiple *visits*). When data reflect different types of visitation counts, it may be more prudent to compare trends, rather than absolute counts.

## Data Availability

Although the dataset of African protected area visitation is self-contained in a single.csv format on Zenodo^80^, end-users may want to transform, summarise, and display the data by recycling the code developed for R version 4.4.0^83^, which is available in the same Zenodo repository^80^. This code script depends on the packages maps^84^ and RColorBrewer^85^ to replicate the analyses shown in Fig. 3.
